# Recent Advances in Biological Functions of Thick Pili in the Cyanobacterium *Synechocystis* sp. PCC 6803

**DOI:** 10.3389/fpls.2020.00241

**Published:** 2020-03-10

**Authors:** Zhuo Chen, Xitong Li, Xiaoming Tan, Yan Zhang, Baoshan Wang

**Affiliations:** ^1^Shandong Provincial Key Laboratory of Plant Stress, College of Life Sciences, Shandong Normal University, Jinan, China; ^2^State Key Laboratory of Biocatalysis and Enzyme Engineering, School of Life Sciences, Hubei University, Wuhan, China; ^3^Biotechnology Research Center, Shandong Academy of Agricultural Sciences, Jinan, China

**Keywords:** thick pili, motility, phototaxis, DNA uptake, *Synechocystis* sp. PCC 6803

## Abstract

Cyanobacteria have evolved various strategies to sense and adapt to biotic and abiotic stresses including active movement. Motility in cyanobacteria utilizing the type IV pili (TFP) is useful to cope with changing environmental conditions. The model cyanobacterium *Synechocystis* sp. PCC 6803 (hereafter named *Synechocystis*) exhibits motility via TFP called thick pili, and uses it to seek out favorable light/nutrition or escape from unfavorable conditions. Recently, a number of studies on *Synechocystis* thick pili have been undertaken. Molecular approaches support the role of the pilin in motility, cell adhesion, metal utilization, and natural competence in *Synechocystis*. This review summarizes the most recent studies on the function of thick pili as well as their formation and regulation in this cyanobacterium.

## Introduction

Cyanobacteria are the only prokaryotes capable of performing oxygenic photosynthesis and still significantly contribute to primary production on a global scale. They are adaptive in a wide range of ecological habitats. Certain species of cyanobacteria were observed to deal with changeable environment via type IV pili (TFP). For example, in the model cyanobacterium *Synechocystis* sp. PCC 6803 (hereafter *Synechocystis*), phototactic motility driven by TFP allows such cyanobacterium to respond to fluctuations in the intensity and spectral quality of light (Yoshihara and Ikeuchi, 2004).

The cells of *Synechocystis* are covered by two distinct types of pili as extracellular appendages. One morphotype is specified as thick pili with an external diameter of 5 nm and a length of more than 2 μm, and another morphotype is defined by thin pili with a diameter of 3–4 nm and a length of less than 1 μm (Bhaya et al., 2000; Yoshihara et al., 2001). Thin pili distribute along the entire cell surface and align in bundles (Yoshihara et al., 2001). So far, genes involved in the formation of thin pili and their roles are unknown. In contrast, *Synechocystis* thick pili have been well dissected.

Thick pili of *Synechocystis*, which belong to TFP, have much in common with that of heterotrophs (Wilde and Mullineaux, 2015). *Synechocystis* cells use thick pili for extension, adhesion to the substrate and retraction to pull the cell across the surface. Multiple motility-related genes have been identified for the function of pili in this model organism (Yoshihara and Ikeuchi, 2004; Schuergers and Wilde, 2015). Besides motility, TFP have been shown to be involved in a range of cellular processes, such as natural transformation (NT) (Bhaya et al., 2000; Yoshihara et al., 2001, 2002), biofilm formation (Chandra et al., 2017; Allen et al., 2019), and metal acquisition (Lamb et al., 2014; Lamb and Hohmann-Marriott, 2017). The biogenesis of thick pili is regulated at multiple levels and has also been studied in *Synechocystis* (Kizawa et al., 2016; Gonçalves et al., 2018; Hu et al., 2018). This review summarizes recent advances on thick pili, including their function, biogenesis and regulation in this cyanobacterium.

## Pilus Apparatuses and Their Encoding Genes

**FIGURE 1:**
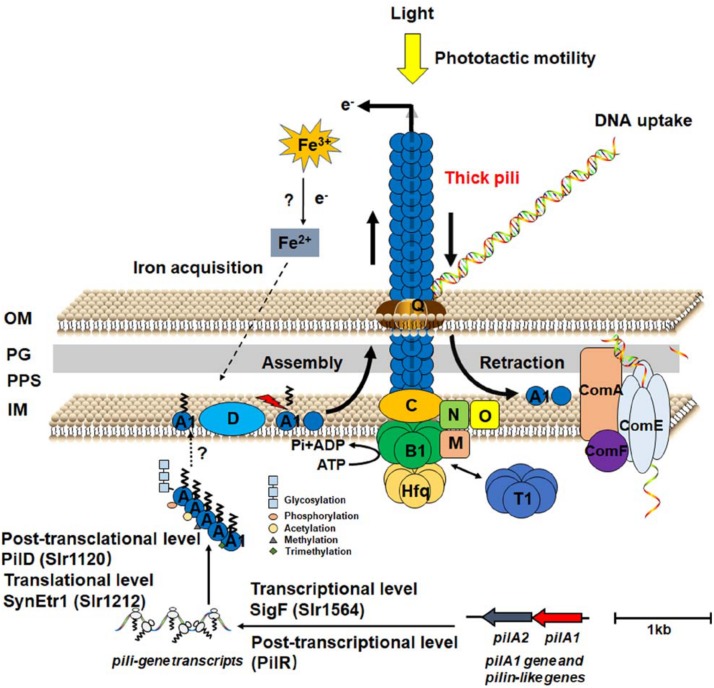
Schematic illustration of the assembly, retraction, function and regulation of thick pili apparatus in *Synechocystis*. A1, B1, C, D, O, M, N, Q, and T1 are short for Pil-related proteins. OM, outer membrane; PG, peptidoglycan; PPS, periplasmic space; IM, inner membrane; SynEtr1, ethylene receptor; SigF, sigma factor; PilR, antisense RNA of PilA11; Hfq, RNA chaperone; ComA/E/F, competence protein.

Thick pilus apparatus is built up with PilA1 and other components for biogenesis and assembly. PilA1 constitutes the major pilin subunit and anchors on the inner membrane, extends across the periplasm space, and/or the outer membrane (Wendt and Pakrasi, 2019). PilA2–PilA8 seem to be dispensable for pilus biogenesis and motility (Bhaya et al., 2000; Yoshihara et al., 2001). Whereas the specific role of these pili-like proteins is unclear. Furthermore, PilD contributes to excising the N-terminal signal peptide and methylation of PilA1 (Linhartová et al., 2014). Assembly of the pilus complex requires two ATPases, PilB, and PilT. The extension motor PilB energizes assembly of thick pili, whereas PilT is required for pilus depolymerization. PilB and PilT are located at the pilus base, while PilC is embedded in the inner membrane (Schuergers and Wilde, 2015). The RNA chaperone Hfq localizes to the pilus base via interaction with PilB1 (Schuergers et al., 2014). PilQ functions as the pore for pilus secretion across the outer membrane. PilMNO proteins are thought to connect the PilQ secretin pore with inner membrane proteins (Schuergers and Wilde, 2015; Figure 1). Besides, there might be other unknown components to be characterized in the pilus apparatus.

## Motility and Phototaxis

Motility, including both gliding and twitching motility, is a frequently observed and beneficial feature among cyanobacteria. The cells of *Synechocystis* sense and shift their location via twitching motility in response to environmental stimuli. TFP are extended by PilB, then adhere to the substrate. Next, pilus retraction is induced by PilT, and cells are pulled across the surface (Burrows, 2012). Thick pilus retraction drags *Synechocystis* cells in a jerky motion across moist surfaces (Burriesci and Bhaya, 2008; Wilde and Mullineaux, 2015). The direction of movement correlates with localization of PilB patches at the inner membrane (Schuergers et al., 2015). The identification of key genes involved in the motility of *Synechocystis* began from the observation of some spontaneous mutants with different motility (Tajima et al., 2011; Kanesaki et al., 2012; Trautmann et al., 2012; Morris et al., 2014; Ding et al., 2015). There are two distinct major groups of *Synechocystis* substrains: the motile PCC and non-motile GT-lineages (Morris et al., 2017). Genes critical for motility and pilus biosynthesis may have mutations among strains, for instance a frameshift mutation in *pilC* (Bhaya et al., 2000), insertion in *spkA*, deletion upstream and within *slr2031*, and/or deletion of a single nucleotide within *slr0322* (Ding et al., 2015). Widespread genomic variations may contribute to the discovery of *Synechocystis* strain-specific mutations related to motility.

Phototactic movement, also known as phototaxis, mediated by thick pili allows *Synechocystis* to move either toward or away from a light source according to light wavelength and intensity. *Synechocystis* harbors a variety of photoreceptors to perceive and respond to the direction, quantity, and quality of illumination (Choi et al., 1999; Ng et al., 2003). Photoreceptors responsible for phototaxis include blue/green light-absorbing proteins PixJ1 and Cph2, blue-light receptor PixD, and UV-A receptor UirS (also known as ethylene receptor, SynEtr1) (Yoshihara et al., 2000; Wilde et al., 2002; Okajima et al., 2005; Song et al., 2011; Sugimoto et al., 2017). Analysis of phototactic movement on low concentration of agar or agarose plates can be achieved at macroscopic and microscopic scales, which represents group behavior and single-cell motility, respectively (Jakob et al., 2017). Individual cells directly and accurately sense the position of light rather than respond to a spatiotemporal gradient in light intensity. During this process, *Synechocystis* cells act as spherical microlenses sensing and moving toward the light source (Schuergers et al., 2016). By fluorescent labeling of thick pili, quantitative analyses of cell tracking indicate asymmetric distribution of cells along the light axis for directional cell motility (Nakane and Nishizaka, 2017). However, the details of *Synechocystis* phototaxis need to be further explored.

## Cell Adhesion

*Synechocystis* may transit from motile states to cell adhesive states, like flocculation and sessile biofilm. These processes include cell-cell and cell-substrate adhesion through cell surface fractions, such as TFP. TFP in *Synechocystis* have recently been revealed to be responsible for biofilm and floc formation (Allen et al., 2019; Conradi et al., 2019). Mutants lacking TFP were unable to aggregate, and loss of *pilC* was shown to significantly reduce biofilm formation and prevent flocculation as well. Except for PilC, the PilB1 and PilT1 proteins are required for flocculation, but PilA1 may be completely absent, indicating the active cycles of thick pilus extension and retraction is dispensable for flocculation. Besides, the minor pilins encoded by the *pilA9-slr2019* operon have been implicated in cell-cell adhesion in flocculation and the processes switching between cell-cell adhesion and cell surface adhesion depend on cyclic AMP (cAMP) levels (Chandra et al., 2017). It was speculated that alteration of surface-attached behavior responsible by TFP might confer *Synechocystis* the resistance to adverse stimuli during the environmental adaptation.

## Natural Competence

Natural transformation is a generally conserved mechanism of horizontal gene transfer among bacteria (Lorenz and Wackernagel, 1994). Natural competence in *Synechocystis* was reported in the late 1980s (Williams, 1988), making this alga as an important model cyanobacterial strain for molecular genetic studies. The process of NT in bacteria mainly includes DNA uptake, transport, processing, and recombination (Johnston et al., 2014). Furthermore, genomic analysis in cyanobacteria has cataloged the genes that are involved in NT post entry of the DNA into the periplasm in a similar manner (Cassier-Chauvat et al., 2016). *Synechocystis* TFP have been proven to be crucial for the uptake of extracellular DNA from the extracellular milieu (Yoshihara et al., 2001). Subsequently, PilQ is found to bind DNA for transport into the periplasm, one strand of the dsDNA is then degraded by an endonuclease, and the other strand is transported across the inner membrane via the competence gene products (Com) ComA (Slr0197), ComE (Sll1929), and ComF (Yoshihara et al., 2001; Nakasugi et al., 2006). After internalization, the ssDNA strand may be bound by recombination mediator proteins, which would recruit recombinases into a complex to promote homologous recombination (Salleh et al., 2019). Nevertheless, many details of these processes are still unclear in *Synechocystis*.

Recently, this conserved mechanism of horizontal gene transfer by TFP competence pili during NT has been revealed in *Vibrio cholerae* (Ellison et al., 2018). The surface pili directly binding to DNA via their tips and then internalizing DNA through retraction could be observed using fluorescent probes. It is likely that similar processes occur during DNA uptake in *Synechocystis*. Besides TFP, NT of *Synechocystis* is also affected by the physiological factors, including phase of cell growth, foreign DNA concentration and incubation time of cells and DNA (Zang et al., 2007). Genome analysis deciphered that pilus structural and assembly proteins and Com proteins are highly conserved across the known species of competent cyanobacteria species (Wendt and Pakrasi, 2019). For instance, the introduction of TFP assembly protein PilN from *Synechococcus elongatus* PCC 7942 into *S*. *elongatus* UTEX 2973 successfully recovered its natural transformability (Li et al., 2018). Whereas, mutants defective in *comF* were not transformable, confirming its role in natural transformation. The transcripts of *comA* in such mutants was not significantly affected, indicating an alternative pathway independent of ComA may exist in *Synechocystis* (Nakasugi et al., 2006). Therefore, to a large extent, the presence of these conserved proteins may explain why some cyanobacterial species are naturally competent whereas others are not.

## Metal Utilization

Being considered as microbial nanowires, TFP may enable metal acquisition for microbial cells and facilitate electron donation to extracellular electron acceptors in some bacteria such as *Shewanella oneidensis* MR–1 (Gorby et al., 2006). Nevertheless, Pirbadian et al. (2014) found that *S. oneidensis* does not produce conductive pili. Recently, it was revealed that microbial nanowires in *G. sulfurreducens* that were earlier thought to be TFP were assembled by polymerized chains of the hexaheme cytochrome OmcS (Wang et al., 2019). Later, Lovley and Walker (2019) evaluate the available evidence on the *in vivo* expression of electrically conductive pili and OmcS filaments and support that both of these two proteins are required for electron transfer in *G. sulfurreducens*. Electrically conductive *Synechocystis* nanowires have also been investigated using scanning tunneling microscopy (Gorby et al., 2006) and subsequently conductive atomic force microscopy (Sure et al., 2015). Further evidences should be provided to determine whether nanowires in this strain are assembled by PilA1. In addition, *Synechocystis* Δ*pilA1* mutants exhibit slower growth rates than wild type on oxidized iron minerals, indicating the role of PilA1 in electron transport to iron oxides. Physiological and spectroscopic data suggested the role of *Synechocystis* PilA1 in oxidization of iron minerals (Lamb et al., 2014), enhancement of manganese acquisition (Lamb and Hohmann-Marriott, 2017) and non-metallic element arsenic deposition (Sure et al., 2016).

## Genes Involved in Pilin Regulation

Comparative genomic analysis clearly showed that genes encoding core components of TFP display high sequence similarity among divergent bacterial groups (Pelicic, 2008). The *Synechocystis* genome contains pilin and pilin-like genes that are organized mainly in polycistronic operons (Kaneko et al., 1996). Table 1 summarizes genes involved in motility, including regulatory pilin genes (Supplementary Figure S1A).

**TABLE 1:** Genes involved in motility in *Synechocystis*.

Gene ID	Gene name	Gene annotation	References
**Signal transduction for pilus assembly and motility**			
*sll0041*	*pixJ1*	Methyl-accepting chemotaxis protein	Yoshihara et al., 2000
*sll0042*	*pixJ2*	Methyl-accepting chemotaxis protein	Yoshihara et al., 2000
*slr1694*	*pixD*	Blue-light using flavin photoreceptor	Schuergers et al., 2015
*slr1212*^*a*,^*^*c*^*	*uirS/SynEtr1^*a*,^^*c*^*	UV-A photosensor	Song et al., 2011
*sll0821*	*cph2*	Phytochrome-like photoreceptor	Park et al., 2000
*slr1143*	*cip1*	Cph2-interacting protein 1	Angerer et al., 2017
*sll0886*	–	Sll0886 protein	Kirik and Babykin, 2008
*slr1693*	*pixE*	PatA subfamily protein	Okajima et al., 2005
*sll0038*	*pixG*	PatA subfamily protein	Yoshihara et al., 2000
*sll0039*	*pixH*	CheY subfamily protein	Yoshihara et al., 2000
*sll0040*	*pixI*	CheW-like protein	Yoshihara et al., 2000
*sll0043*	*pixL*	CheA-like protein	Yoshihara et al., 2000
*sll0058*	*dnaK1*	Charperone	Bhaya et al., 2001
*slr1214*	*lsiR*	PatA-type regulator	Song et al., 2011
*slr1213*	*uirR*	Response regulator	Song et al., 2011
*sll1371*	*sycrp1*	cAMP receptor	Yoshimura et al., 2000
*sll1924*	*sycrp2*	cAMP receptor	Song et al., 2018
*slr0895*	*prqR*	DNA-binding transcriptional regulator	Kirik et al., 2008
*sll1626*^*a*^	*lexA*^*a*^	Transcription regulator LexA	Kamei et al., 2001a
*slr1564*^*a*^	*sigF*^*a*^	RNA polymerase sigma-37	Bhaya et al., 1999
*sll1575*	*spkA*	Ser/Thr protein kinase	Kamei et al., 2001b
*slr1697*	*spkB*	Ser/Thr protein kinase	Kamei et al., 2002
*slr1443*^*e*^	*spkE^*e*^*	Ser/Thr protein kinase-like protein E	Kim et al., 2004
*slr1991*	*cya1*	Adenylate cyclase	Terauchi and Ohmori, 1999
*ssr3341*^*b*^	*hfq^*b*^*	RNA chaperone	Dienst et al., 2008
*sll0183*	–	Sll0183 protein	Bhaya et al., 2001
*sll0301*	–	Sll0301 protein	Bhaya et al., 2001
*slr0358*	–	Slr0358 protein	Bhaya et al., 2001
*sll0414*	–	Sll0414 protein	Bhaya et al., 2001
*sll0415*	–	ABC-transporter	Bhaya et al., 2001
*sll0564*	–	Sll0564 protein	Bhaya et al., 2001
*sll0565*	–	Sll0565 protein	Bhaya et al., 2001
*sll0899*^*e*^	–	Bifunctional protein GlmU	Kim et al., 2009
*sll0141*^*e*^	–	Sll0141 protein	Gonçalves et al., 2018
*sll0180*^*e*^	–	Sll0180 protein	Gonçalves et al., 2018
*slr0369*^*e*^	–	Cation or drug efflux system protein	Gonçalves et al., 2018
*sll1384*	–	Sll1384 protein	Chen and Xu, 2009
*slr1964*	*frp*	Fluorescence recovery protein	Bhaya et al., 2001
*slr1204*^*d*,^*^*e*^ sll1679*^*d*,^*^*e*^ sll1427*^*d*,^*^*e*^*	*htrA*^*d*,^*^*e*^ degQ*^*d*,^*^*e*^ degS*^*d*,^*^*e*^*	Deg proteases	Barker et al., 2006
–	*PilR*^*b*^	Antisense RNA of *pilA11*	Hu et al., 2018
*slr2031*	–	Sigma factor SibG regulation protein RsbU	Kamei et al., 1998
*slr0388*	*comF*	Competence-related protein	Nakasugi et al., 2006
**Pilus biogenesis and assembly**			
*sll1694*	*pilA1*	Pilin	Yoshihara et al., 2001
*sll1695*	*pilA2*	Pilin-like protein	Bhaya et al., 1999
*slr1456*	*pilA4*	Pilin-like protein	Yoshihara et al., 2001
*slr1928*	*pilA5*	Pilin-like protein	Yoshihara et al., 2001
*slr1929*	*pilA6*	Pilin-like protein	Yoshihara et al., 2001
*slr1930*	*pilA7*	Pilin-like protein	Yoshihara et al., 2001
*slr1931*	*pilA8*	Pilin-like protein	Yoshihara et al., 2001
*slr2015*	*pilA9*	Pilin-like protein	Yoshimura et al., 2002a
*slr2016*	*pilA10*	Pilin-like protein	Yoshimura et al., 2002a
*slr2017*	*pilA11*	Pilin-like protein	Yoshimura et al., 2002a
*slr2018*	*pilA12*	Pilin-like protein	Yoshimura et al., 2002a
*slr0063*	*pilB1*	Pilus assembly	Yoshihara et al., 2001
*slr0079*	*GspE/PulE*	Pilus assembly pathway ATPase	Yoshihara et al., 2001
*slr0162/slr0163*	*pilC*	Inner membrane protein	Bhaya et al., 2001
*slr1120*^*e*^	*pilD*^*e*^	Prepilin peptidase/N-methylase	Bhaya et al., 2001
*slr1041*	*pilG*	PatA-like protein	Yoshihara et al., 2002
*slr1042*	*pilH*	CheY-like protein	Yoshihara et al., 2002
*slr1043*	*pilI*	CheW-like protein	Yoshihara et al., 2002
*slr1044*^*a*^	*pilJ/ctr1^*a*^*	Methyl-accepting chemotaxis protein	Yoshihara et al., 2002
*slr0073*	*pilL-N*	CheA-like protein	Yoshihara et al., 2002
*slr0322*	*pilL-C*	CheA-like protein	Yoshihara et al., 2002
*slr1274*	*pilM*	Pilin pore complex	Yoshihara et al., 2001
*slr1275*	*pilN*	Pilin pore complex	Yoshihara et al., 2001
*slr1276*	*pilO*	Pilin pore complex	Yoshihara et al., 2001
*slr1277*	*pilQ*	Pilin secretin pore	Yoshihara et al., 2001
*slr0161*^*a*^	*pilT1*^*a*^	Pilus assembly	Bhaya et al., 2001
*sll1533*	*pilT2*	Twitching mobility protein	Bhaya et al., 2000
*sll1107*	–	TFP biogenesis protein PilI homolog	Singh et al., 2006

*The superscript lowercase letters of a, b, c, d, and e represent genes involved in transcriptional, post-transcriptional, translational, protein stability, post-translational levels in the regulation of pilin, and/or pilin-like proteins, respectively.*

To date, several genes reported to regulate the expression of *pilA1* at the transcriptional level have been studied *ctr1* (*slr1044*), which encodes a putative methyl-accepting chemotaxis protein (MCP) (Chung et al., 2001); *sigF* (*slr1564*), which encodes an alternative sigma factor (Bhaya et al., 1999); *pilT1* (*slr0161*), which is responsible for pilus depolymerization (Bhaya et al., 2000); *lexA* (*sll1626*), which is involved in cell motility (Kamei et al., 2001a). Of these, Ctr1 protein functions as a transducer, SigF and LexA may play roles as transcriptional factors that positively regulates the expression of *pilA1* (Asayama and Imamura, 2008; Kizawa et al., 2016). While the *pilA1* transcript influenced by PilT1 has not been revealed.

In addition, the RNA chaperone Hfq (Ssr3341) was predicted to play important roles in phototaxis in post-transcriptional regulation in *Synechocystis* (Schuergers et al., 2014). The antisense RNA PilR acts as a direct negative regulator of PilA11 and cell motility (Hu et al., 2018). Disruption in the second ethylene-binding domain of ethylene receptor, named SynEtr1ΔTM2 cells, showed a large increase in both transcriptional and translational levels of PilA1 (Lacey and Binder, 2016). The Δ*Deg* (*slr1204/sll1679/sll1427*) mutant appeared to be hyperpiliated with thick pili, indicating the regulation of PilA1 stability by Deg proteases (Barker et al., 2006).

Moreover, post-translational modifications (PTMs) have been characterized as one of the most important factors in *Synechocystis* pilus function over the past two decades. Various PTMs of pilin in *Synechocystis*, have also been proposed or verified to be important factors in phototactic movement (Sergeyenko and Los, 2000; Kim et al., 2004, 2009, 2011). For instance, Kamei et al. (2001b) found that six Serine/Threonine protein kinases participate in cell motility control, and phosphorylation participates in the phototactic movement process. Later, it was interesting to identify pilin phosphorylated at Serine 59 (Chen et al., 2015) and acetylated at lysine 58 (Mo et al., 2015), implying cross-talk between serine phosphorylation and lysine acetylation. PilD (Slr1120) has been predicted to be responsible for pre-pilin leader peptide cleavage as well as N-terminal methylation (Bhaya et al., 2001). PilD was also identified to play a role in PilA1 glycosylation (Linhartová et al., 2014). Pilin has been confirmed to be glycosylated in *Neisseria* (Chamot-Rooke et al., 2007) and *Pseudomonas* (Voisin et al., 2007), and several pilin-glycosylation genes have also been characterized in *Synechocystis.* Notably, Kim et al. (2011) discovered trimethylation at the C-terminal lysine and O-glycosylation within the pilus peptide in *Synechocystis*, indicating an indispensable role of PTMs for pilus assembly and pilus-mediated motility. *sll0899* is involved in O-glycosylation between amino acids 67 and 75 in pilin and inactivation of *sll0899* produces increased molecular mass of pilins (Kim et al., 2009). *sll0141*, *sll0180*, and *slr0369* genes, which encoded putative inner membrane translocase components of TolC-mediated secretion, are also responsible for pilus glycosylation. Nevertheless, motility assays confirmed that Sll0141 and Slr0369 are not essential for motility (Gonçalves et al., 2018). The three *Deg* mutants with less fucose in cells were found to have impaired motility, implying impairment of PilA1 glycosylation (Cheregi et al., 2015). Additionally, other types of PTMs and functional associations with PilA1 may exist in *Synechocystis*, similar to other bacteria (Stimson et al., 1996; Naessan et al., 2008). Moreover, it will be more meaningful to dissect the specific role of such modification sites on the mature pili in this model organism.

## Motility Signaling Pathway

Currently, increasing evidence suggest that *Synechocystis* motility is associated with several signaling pathways (Supplementary Figure S1B), for example the cAMP and/or cyclic-di-GMP-mediated pathway (Bhaya et al., 2006; Yoshimura et al., 2010; Savakis et al., 2012; Xie et al., 2018). Two cAMP receptor-like proteins, named as Sycrp2 and Sycrp1, are known to be involved in twitching motility (Song et al., 2018). Sycrp1 has binding affinity for cAMP and directly binds to the upstream region of *slr1667*, and positively regulates the expression of *pilA9*–*pilA10*–*pilA11*–*slr2018* gene cluster (Yoshimura et al., 2002a). Sycrp2 does not bind cAMP (Yoshimura et al., 2000, 2002b) but may interact and work with Sycrp1 without functional redundancy (Song et al., 2018). Photoreceptor Cph2 with the GGDEF domain acts as a blue-light triggered c-di-GMP producer and thereby inhibits cell motility in blue light. Furthermore, Cph2 modulates motility by interacting with Cip1 (Slr1143, Cph2-interacting protein 1) under red light (Angerer et al., 2017). MCP–CheA–CheY systems include *sll0038*–*sll0043*, *slr1041*–*slr1044* and *slr0322*, *slr0073*, *sll1291*–*sll1296* three gene clusters (Chung et al., 2001; Yoshihara et al., 2002). Jakob et al. (2019) have confirmed the direct interaction of the PixD-PixE complex with PilB1, suggesting that blue-light dependent negative phototaxis is controlled by the PixD-PixE signal transduction system. Using a computer-assisted video microscope motion analysis system, researchers found that Ca^2+^ plays a significant role in regulating *Synechocystis* photo-orientation and motility (Moon et al., 2004).

Additionally, histidine kinases (Hik18, Hik36, and Hik43) were predicted or demonstrated to be involved in phototaxis (Xu and Wang, 2019). Hik18 regulates positive phototaxis by suppressing pilus biosynthesis and expression of regulatory genes through the interplay with positive phototaxis/motility two-component proteins (Shin et al., 2008). An ethylene-responsive signaling pathway affecting phototaxis was also characterized in *Synechocystis* (Kuchmina et al., 2017). Endogenous ethylene produced by heterologous expression of the *Pseudomonas syringae* ethylene-forming enzyme accelerates positive phototaxis. Ethylene mainly inactivates transcription from the csiR1/lsiR promoter. This promoter is under the control of UirS and its response regulator UirR. *Synechocystis* might use ethylene as an environmental signal in aquatic environments (Lacey and Binder, 2016). Further details of signal transduction regulating motility in *Synechocystis* remain to be explored.

## Perspective

There is substantial investigation of biological function of the thick pili in the model cyanobacterium *Synechocystis*. For instance, emerging evidence suggest the signaling pathway of motility is mediated by thick pili, which actively sense and respond to several environmental conditions. Apart from the subunit of the thick pilus apparatus, some regulatory proteins and small RNAs involved in these processes are likely to directly or indirectly influence thick pilus biogenesis and function. To uncover these regulation of pilus genes or their coding products, we may resort to mutant library and high-throughput “-omics” to facilitate elucidation of pilus-involved signaling pathways. In addition, thick pili of *Synechocystis* may also be significantly used in large-scale applications for biofuel biotechnology owing to their characteristics of biofilm formation and adhesion, which will render cell immobilization, biomass harvesting, and product purification more convenient. Although there is a contentious debate over microorganisms which can produce conductive pili, the studies on the *Synechocystis* thick pili may also have practical applications in the sustainable composite materials, and further bioavailability of metals in the future.

## Author Contributions

ZC designed and wrote the manuscript. XL revised the figures. YZ, XT, and BW made major revisions of the manuscript.

## Conflict of Interest

The authors declare that the research was conducted in the absence of any commercial or financial relationships that could be construed as a potential conflict of interest.

## Supplementary Material

FIGURE S1 Schematic illustration of genes involved in pilin regulation at different levels **(A)** and three potential signal pathways involved in motility in *Synechocystis* **(B)**.
