# Supplementary figures and images for: Recent Advances in Biological Functions of Thick Pili in the Cyanobacterium Synechocystis sp. PCC 6803
Source: Front Plant Sci. 2020 Mar 10;11:241. doi: 10.3389/fpls.2020.00241 (PMC7076178; doi:10.3389/fpls.2020.00241)

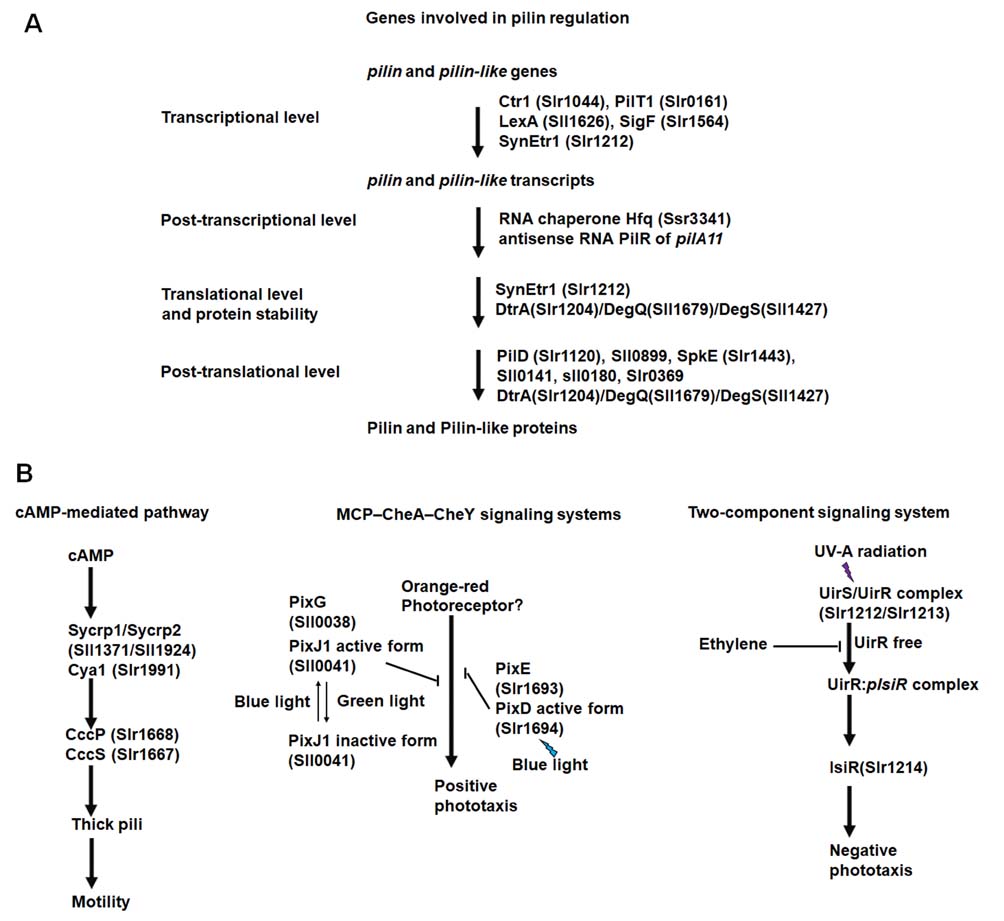

Supplement: FIGURE S1 — Schematic illustration of genes involved in pilin regulation at different levels (A) and three potential signal pathways involved in motility in Synechocystis (B). [file Image_1.jpg]
